# Hospital and Institutionalisation Care Costs after Limb and Visceral Ischaemia Benchmarked Against Stroke: Long-Term Results of a Population Based Cohort Study

**DOI:** 10.1016/j.ejvs.2018.03.007

**Published:** 2018-08

**Authors:** Ramon Luengo-Fernandez, Dominic P.J. Howard, Kathleen G. Nichol, Emily Dobell, Peter M. Rothwell, the Oxford Vascular Study

**Affiliations:** a Centre for Prevention of Stroke and Dementia, Nuffield Department of Clinical Neurosciences, University of Oxford, UK; b Department of Vascular Surgery, Oxford University Hospitals NHS Foundation Trust, UK; c Oxford University Hospitals NHS Foundation Trust, UK; d Oxford School of Public Health, Nuffield Department of Population Health, University of Oxford, UK

**Keywords:** Costs, Peripheral vascular disease, Stroke

## Abstract

**Objective/background:**

There are few published data on the acute care or long-term costs after acute/critical limb or visceral ischaemia (ACLVI) events. Using data from patients with acute events in a population based incidence study (Oxford Vascular Study), the present study aimed to determine the long-term costs after an ACLVI event.

**Methods:**

All patients with first ever incident ACLVI from 2002 to 2012 were included. Analysis was based on follow up until January 2017, with all patients having full 5 year follow up. Multivariate regressions were used to assess baseline and subsequent predictors of total 5 year hospital care costs. Overall costs after an ACLVI event were benchmarked against those after stroke in the same population, during the same period.

**Results:**

Among 351 patients with an ACLVI event, mean 5 year total care costs were €35,211 (SD 50,500), of which €6443 (18%) were due to long-term institutionalisation. Costs differed by type of event (acute visceral ischaemia €16,476; acute limb ischaemia €24,437; critical limb ischaemia €46,281; *p* < 0.001). Results of the multivariate analyses showed that patients with diabetes and those undergoing above knee amputations incurred additional costs of €11,804 (*p* = 0.014) and €25,692 (*p* < 0.001), respectively. Five year hospital care costs after an ACLVI event were significantly higher than after stroke (€28,768 vs. €22,623; *p* = 0.004), but similar after including long-term costs of institutionalisation (€35,211 vs. €35,391; *p* = 0.957).

**Conclusion:**

Long-term care costs after an ACLVI event are considerable, especially after critical limb ischaemia. Hospital care costs were significantly higher than for stroke over the long term, and were similar after inclusion of costs of institutionalisation.

**What this paper adds**

This is the first prospective, population based study of acute/critical limb or visceral ischaemia (ACLVI) events in a population of 92,728 in Oxfordshire, UK. The study shows that the long-term costs after such events are substantial, and that costs vary considerably across type of event. When compared with stroke, hospital care costs are significantly higher after an acute visceral and peripheral arterial event, and similar after inclusion of institutionalisation costs. The present data will be useful to other researchers to perform comparisons in attempts to better understand the likely economic consequences of ACLVI events in their own setting.

## Introduction

Cardiovascular disease is the leading cause of death and disability, costing the US and European Union healthcare systems $196 billion and €111 billion, respectively.^1^, ^2^ Therefore, there has been much research interest to prevent, diagnose, and treat vascular disease.

However, although peripheral arterial disease (PAD) has a poor prognosis,^3^ it has been neglected in terms of research,^4^ and there are therefore a paucity of data evaluating the economic impact of PAD on healthcare systems. Although a number of studies have been published assessing the care costs of PAD, these have tended to be based on hospital coding data of diagnosis or interventions;^5^, ^6^, ^7^ concentrated only on patients with diabetes or specific interventions,^8^, ^9^ more stable PAD;^10^ or randomised controlled trials with stringent inclusion criteria;^11^ and have tended to omit long-term institutionalisation care costs. This lack of economic evidence on PAD outcome therefore limits comparisons of outcome and cost between this and other conditions, which in turn reduces the ability to make decisions about the relative funding requirements for service provision and research.

Using data from patients with an acute/critical limb or visceral ischaemia (ACLVI) event in a population based incidence study (Oxford Vascular Study [OXVASC]) ascertained between 2002 and 2012, the present study aimed to reliably determine the absolute long-term costs and their baseline and subsequent predictors. In addition, given that the costs of other vascular disease, in this case stroke, have been estimated in the same population and using the same methodology,^12^, ^13^ there is a unique opportunity to benchmark the long-term costs of these acute events against stroke.

## Materials and Methods

### The Oxford Vascular Study

The Oxford Vascular Study (OXVASC) population comprises all individuals (2002–2012 average = 92,728) registered in nine general practices across Oxfordshire, UK. The study methods have been described elsewhere,^3^, ^14^ and are also reported in the online supplementary appendix. Briefly, patient registration began on April 2002, and is ongoing. Patients in whom an ACLVI event was suspected were ascertained. An ACLVI event in this study is defined as any arterial event that affected a limb or an organ other than the heart or the brain/eye and led to hospital assessment or admission or caused death in the community. Acute limb ischaemia (ALI), critical limb ischaemia (CLI), and acute visceral ischaemia (AVI) are included within this definition. Ascertainment was undertaken using multiple overlapping methods of pursuit and considered for inclusion, including prospective daily searches for acute events in hospital and supplemented by searches of discharge and primary care diagnostic coding data. OXVASC was approved by the local research ethics committee.

All diagnoses were reviewed by a vascular surgeon. Cardiovascular examination included assessment of the peripheral pulses, Buerger's test,^15^ and absolute ankle pressure and ankle brachial pressure index recordings. For patients with incompressible ankle signals, pressures were estimated by pole test. For patients in whom clinical vascular assessment was not possible by the study clinician before urgent revascularisation or death, the assessments made by the admitting clinician were used. If a patient died before assessment or was identified only by cold pursuit, eyewitness accounts were obtained, and relevant records reviewed. If death occurred outside the hospital or before investigation, autopsy results were reviewed. Clinical details were sought from primary care physicians or other clinicians on all deaths resulting from a possible vascular cause.

All patients with a first ever incident ACLVI event from April 1, 2002 to March 31, 2012 from the study registered practices were included and followed-up. ALI was defined as an arterial event of sudden onset and <2 weeks in duration resulting in symptomatic limb ischaemia. AVI was defined as acute arterial events of sudden onset and <2 weeks in duration resulting in symptomatic visceral ischaemia (including bowel, liver, spleen, and renal end organ compromise). CLI was defined as an event with symptoms present for >2 weeks with ischaemic rest pain or tissue loss of sufficient severity to warrant urgent hospital admission and thought to be secondary to large or small vessel arterial disease. History of stable PAD was defined as symptomatic PAD without prior ACLVI history or emergency intervention. These patients typically have intermittent claudication (calf cramping) with any pain at rest or ulceration.

All surviving patients were followed up by the following methods: 1) 6 month face to face follow up; 2) primary care records; 3) ongoing study ascertainment of hospital contacts of study participants and the wider study population; 4) routine review of administrative hospital and centralised care records; 5) mortality records; and 6) review of Coroner's office death reports for deaths outside hospital or during surgical procedures. If a vascular event was suspected, the patient was reassessed by a study physician. All patients had mortality follow up.

### Resource use and unit costs

The following healthcare costs were included: accident and emergency visits, emergency transport by ambulance, inpatient care stays, day cases, and outpatient care visits. In terms of social care, costs associated with stays in a nursing or residential care home were included. Hospital care resource use was obtained from computerised patient hospital records from the date of first ever incident ACLVI event until death or 5 years after the event, whichever was first.

Patients' centralised Hospital Episodes Statistics (HES) records, as well as those from the Oxford University Hospitals NHS Foundation Trust (which is made up of 4 hospitals) and nine Oxfordshire community hospitals, were reviewed. Information on any accident and emergency visit, emergency transport, outpatient care visit, day case, or hospitalisation was obtained. For each hospital admission, information was recorded on the date of admission and discharge, including the dates of transfers between different specialty wards. Hospitalisations during which patients were admitted and discharged on the same day were classified as day cases. Hospital admissions resulting from the incident ACLVI event were defined as the first admission occurring within 7 days of the event. Subsequent admissions resulting from an ACLVI event were defined as: 1) admissions within 7 days of any subsequent ACLVI event; 2) admissions within 7 days of any subsequent intervention or investigation because of ACLVI events; and 3) hospital admissions (e.g. hospital transfers) immediately after incident or subsequent ACLVI events, or because of interventions or investigations. For the purposes of this study, long-term institutionalisation was defined as admission into a nursing or residential care home. Date of institutionalisation was obtained from patients' study, primary, and hospital care records.

Regardless of when hospital resources were consumed by patients, all resource use was priced using 2015/16 unit costs. All healthcare unit costs were derived from the schedule of National Health Service reference costs.^16^ The unit costs used to price inpatient resource use also included the costs of treatment, investigations, and interventions typically performed in each specialty ward. Institutionalisation was costed as the cost per week in a private nursing home, which in 2016 was £795 (€836) per week.^17^ Institutionalisation costs for patients already in long-term care at the time of the event were not included in the analysis.

In the main analysis, costs incurred by patients after the first year have not been discounted to present value terms. However, sensitivity analyses are presented in which costs incurred after the first year were discounted using a 3.5% annual rate to reflect current UK recommendations (Table S1).

As part of the analysis, the 5 year care costs after an ACLVI event were compared with those after stroke,^12^, ^13^ with the costs of both conditions being obtained from the same patient population and using the same methodology and unit costs.

All costs were converted from UK pounds sterling (£) to Euros (€), using the rate of purchasing power parities in 2016 (€1 is equal to £0.95).^18^

### Statistical analysis

Time to institutionalisation was defined as the difference between the date on which the incident ACLVI event occurred and the date of admission into a nursing or residential care home. The 5 year risk of institutionalisation in a long-term care home was evaluated using Kaplan–Meier techniques, adjusted for mortality. Statistically significant differences in risk between subgroups were assessed using Cox's proportional hazards model.

All 5 year costs are presented as means together with SD and 95% CI under the Central Limit Theorem hypothesis. Given the high 5 year mortality after an ACLVI event,^3^, ^19^ assessments were made of how costs varied over each of the 5 years of follow up:

• All cases, in which mean annual costs for all patients, including patients who died, are presented;
• Surviving available cases, in which mean annual costs for surviving patients with complete data for that year are presented; and
• Only 5 year surviving cases, in which mean annual costs for 5 year survivors only are presented.

The main predictors of hospital care costs at 5 years were assessed. By means of a univariate analysis, the importance was assessed of each baseline variable at determining hospital costs after the incident ACLVI event. The baseline variables included were: age; gender; history of each of smoking, diabetes mellitus, hypertension, hyperlipidaemia, atrial fibrillation, heart failure, coronary artery disease, previous stroke or TIA, and stable PAD; type of ACLVI event (i.e. ALI, AVI or CLI); severity of ischaemia on admission; and deprivation (as measured using the index of multiple deprivation by postcode of residence at event onset). For all continuous variables (age and index of multiple deprivation), appropriate cut off points were used to stratify patients. All comparisons between groups were performed using two tailed Student *t* test or analysis of variance to examine the differences between/among stratified groups. Using the same baseline variables, a generalised linear gamma model with log identity was used to assess the independent baseline predictors of total costs after the event.

In addition, using both uni- and multivariate analysis, the impact of subsequent ACLVI events and surgical and endovascular interventions was assessed on hospital costs. However, as many of these interventions are not performed in patients with AVI, for a more meaningful interpretation of these costs, only patients with limb ischaemia were included (i.e. ALI and CLI).

A two tailed Student *t* test was used to compare differences in hospital, long-term institutionalisation and overall mean costs between patients with an ACLVI event and patients with stroke. Statistical significance was set at *p* < 0.050. Model specification was tested using the Ramsey reset test, a general specification test for the linear regression model used to test whether non-linear combinations of the fitted values helped explain costs.

## Results

### Study sample

Between April 2002 and March 2012, 351 patients had a first ever incident ACLVI event, of which 81 (23%) were ALI, 71 (20%) AVI, and 199 (57%) CLI. The mean age was 76 (SD 12) years, with males accounting for half the sample (*n* = 174). Half the sample (*n* = 177, 50%) had a confirmed history of stable PAD, and a significant proportion of patients had other previous symptomatic vascular disease (Table S2). Of the 351 patients with an incident ACLVI event, 113 (32%) were alive at 5 years.

### Long-term total care costs after an ACLVI event

**Figure 1:**
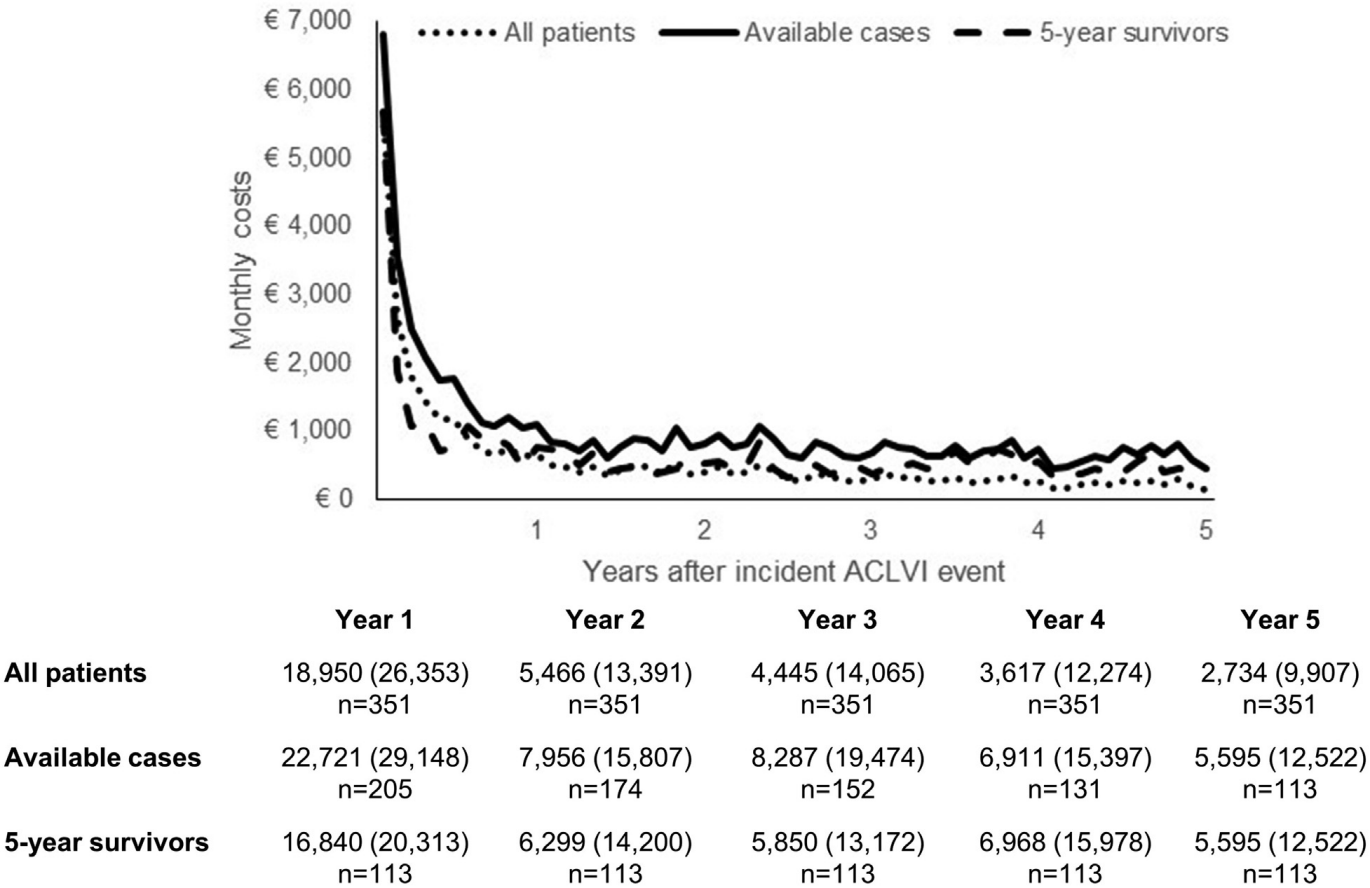
Five year total care costs (€) after an incident ACLVI event.

For all 351 patients with an ACLVI event, the mean 5 year total care costs after the event were €35,211 (95% CI 29,910–40,513), of which 18% (€6443) were due to long-term institutionalisation admission and 72% (€28,768) were hospital care costs (Table 1). As shown in Fig. 1, over half of the mean total care costs incurred by the 351 patients with an ACLVI event (€18,950, 54%) were incurred during the first year after the event, with costs gradually decreasing over time because of patient mortality. For the 113 patients who survived past 5 years, mean total care costs were €41,522 (SD 62,349), with annual costs remaining constant after the first year (Fig. 1).

**Table 1:** Five year mean care costs (€, SD) after incident ACLVI event.

	Emergency transport	Accident & Emergency	Day cases	Inpatient stays	Outpatient visits	Total hospital costs	Long-term institutionalisation	Total care costs (95% CI)
All events	252 (372)	174 (253)	1091 (3037)	25,068 (32,944)	2183 (4016)	28,768 (34,859)	6443 (30,468)	35,211 (29,910–40,513)
ALI	208 (320)	144 (219)	818 (1320)	16,614 (30,140)	1595 (2254)	19,378 (31,203)	5059 (29,258)	24,437 (14,377–34,497)
AVI	215 (335)	141 (208)	552 (1290)	6938 (13,270)	542 (1190)	8389 (14,695)	8087 (39,279)	16,476 (5265–27,687)
CLI	283 (401)	198 (277)	1394 (3845)	34,977 (35,262)	3008 (4916)	39,861 (37,008)	6420 (27,348)	46,281 (39,178–53,385)

ANOVA test for differences in total costs in ACLVI event groups at 5 years < 0.0001.

**Figure 2:**
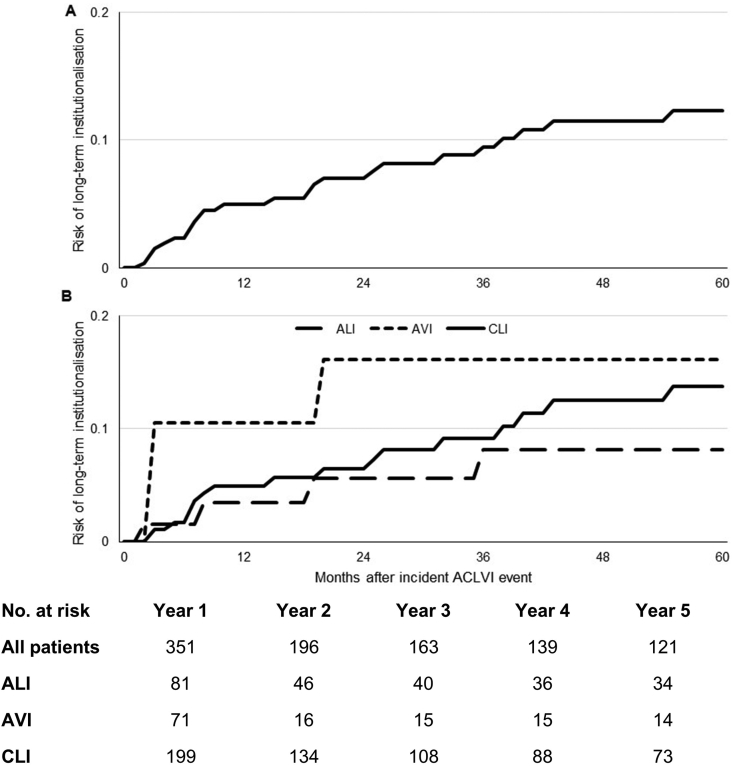
Five year risk of institutionalisation in (A) all patients with an incident ACLVI event; and (B) patients with ALI, AVI, and CLI.

By ACLVI event type, 5 year mean total costs were: €16,476 (95% CI 5265–27,687) for AVI; €24,437 (14,377–34,497) for ALI; and €46,281 (39,178–53,385) for CLI (difference between groups, *p* < 0.0001). Although patients with AVI had the lowest overall 5 year costs, they had the highest long-term institutionalisation costs, with half of all costs (49%; €8087) incurred from institutionalisation, with AVI patients facing a higher risk of institutionalisation in the first 6 months after an incident ACLVI event (11% vs. 2% for ALI and CLI, Fig. 2). Differences in 5 year total costs between ACLVI event types were mainly explained by differences in inpatient hospital stay costs: €6938 (13,270) for AVI; €16,614 (30,140) for ALI; and €34,977 (35,262) for CLI (Table 1). This reflected the differing number of days patients spent in hospital over the 5 years following the incident ACLVI event: 13 days for AVI; 37 days for ALI; and 76 days for CLI (*p* < 0.0001, Table 2).

**Table 2:** Five year hospital care resource use after incident ACLVI event.

	All	ALI	AVI	CLI
Mean encounters per patient per follow up year (standard error)				
A&E visits	0.49 (0.02)	0.38 (0.04)	0.78 (0.09)	0.49 (0.03)
Day cases	1.20 (0.04)	0.42 (0.04)	0.72 (0.09)	1.58 (0.05)
Hospital inpatient admissions	1.44 (0.04)	0.98 (0.07)	1.01 (0.11)	1.69 (0.06)
Outpatient visits	8.35 (0.10)	4.51 (0.15)	3.14 (0.19)	10.69 (0.14)
Mean days in hospital (SD)				
Days because of incident ACLVI	9 (14)	9 (13)	6 (10)	10 (15)
Subsequent admissions				
Because of ACLVI	24 (52)	15 (45)	1 (1)	37 (60)
Other reasons	21 (40)	14 (34)	6 (20)	30 (46)
Total days in hospital	54 (76)	37 (69)	13 (25)	76 (83)

**Figure 3:**
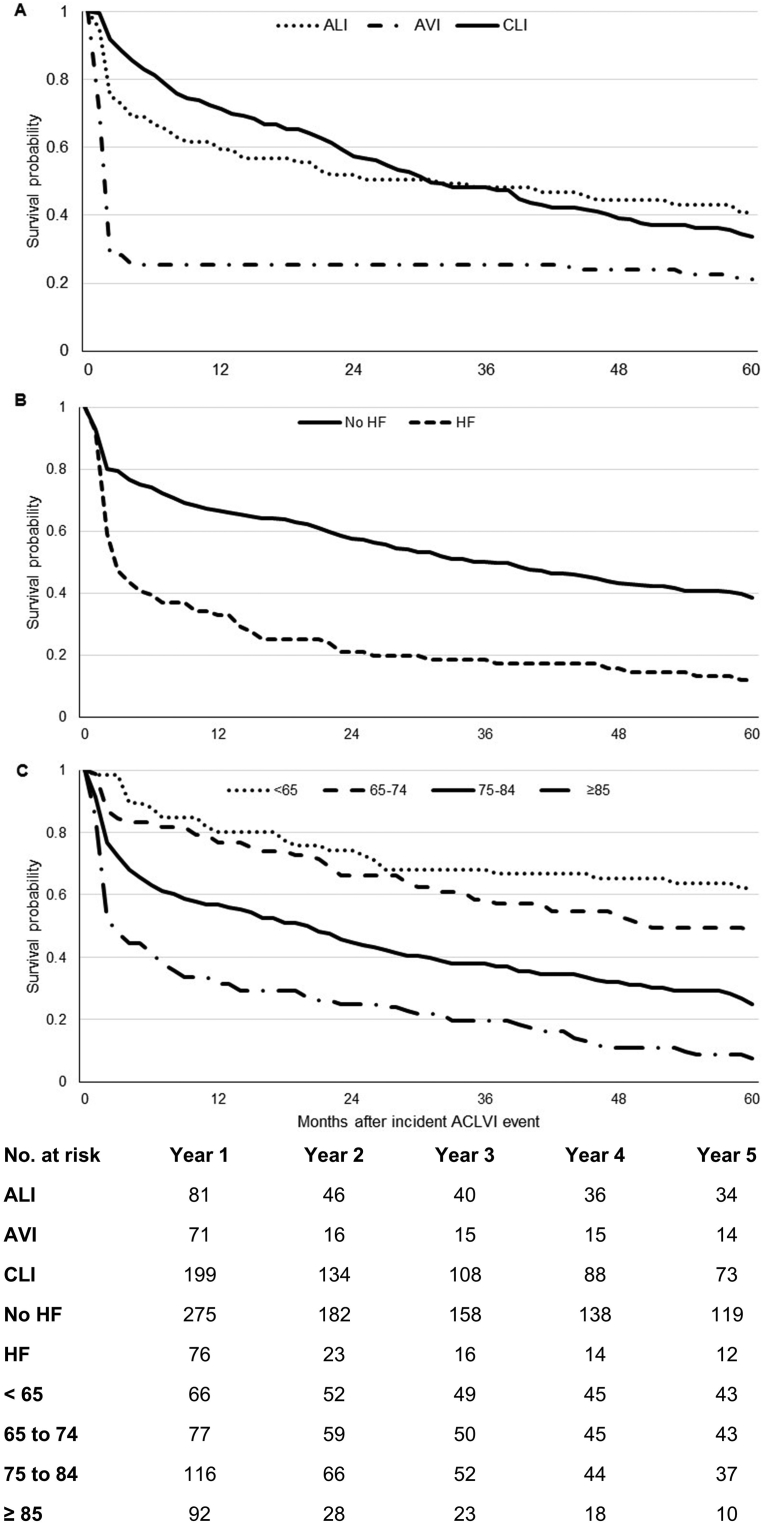
Overall 5 year survival after an incident ACLVI event by (A) type of event; (B) history of heart failure; and (C) age.

**Figure 4:**
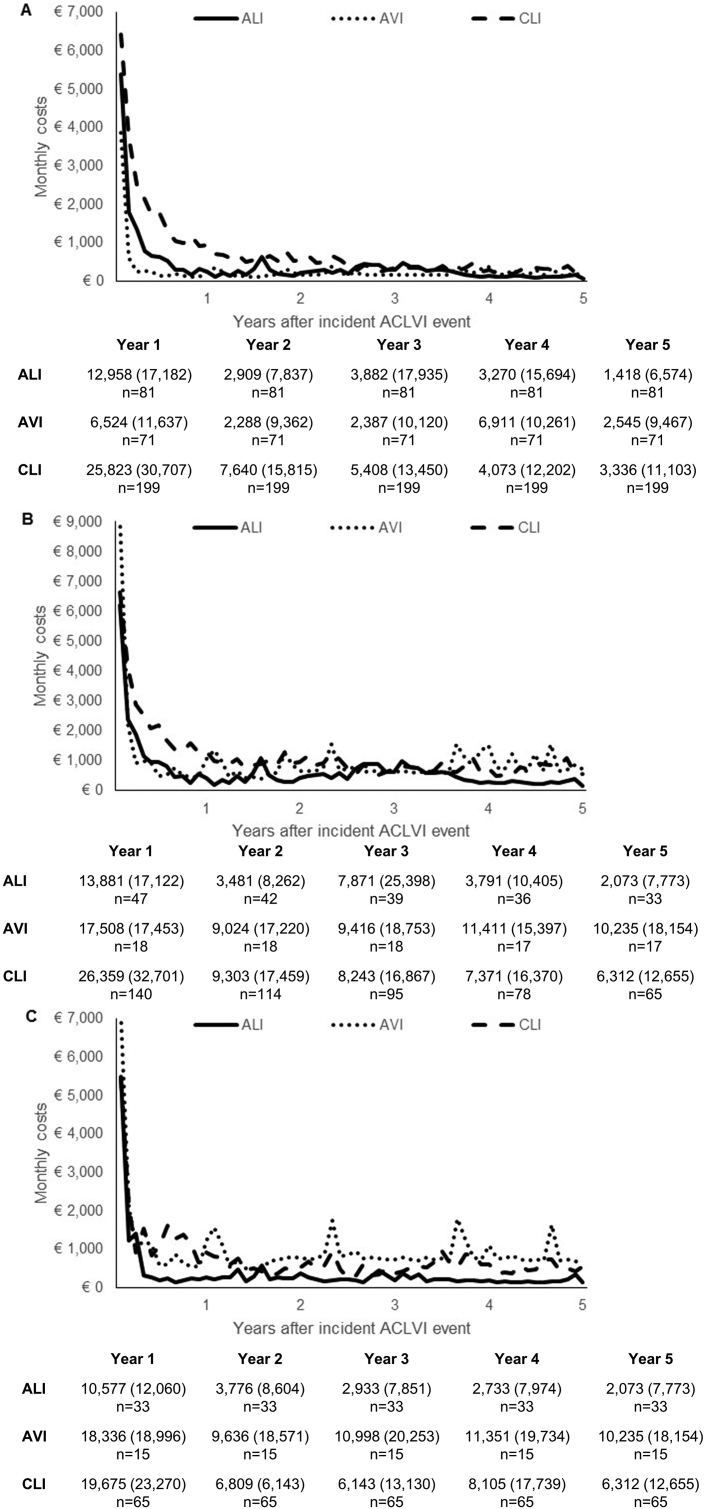
Five year total care costs (€) after ALI, AVI, and CLI for (A) all cases; (B) surviving available cases; and (C) 5 year survivors.

Total care costs were greatly correlated with mortality. Patients with AVI, who had the lowest overall costs, had the highest rates of early mortality, with 6 month mortality rates being 75% (*n* = 53) as opposed to 33% (*n* = 27) for ALI and 21% (*n* = 42) for CLI (*p* < 0.001), who had the highest overall costs. Overall, 5 year life expectancy was 1.25 years after AVI; 2.62 years after ALI; and 2.76 after CLI (Fig. 3), with that for overall ACLVI being 2.42 years. In effect, although AVI patients had the lowest per patient costs when averaged across all patients, among 5 year survivors, AVI patients incurred the highest mean costs (€60,564, SD 90,127), with surviving CLI and ALI patients incurring 5 year costs of €47,044 (62,367) and €22,093 (40,639), respectively (differences across groups, *p* = 0.077, Fig. 4).

### Predictors of long-term hospital costs after an ACLVI event

#### Baseline predictors

Univariate analysis showed that patients with history of stable PAD incurred significantly higher hospital costs over 5 years (€35,248 vs. €22,176, *p* = 0.0004), as did patients with diabetes mellitus (€47,799 vs. €21,103, *p* < 0.0001) and hyperlipidaemia (€35,939 vs. €22,496, *p* = 0.0003; Table S3), mainly reflecting the higher overall costs of CLI. There were also significant differences in total costs according to age and deprivation.

As with the univariate analysis, ACLVI event type was a significant independent predictor of total 5 year costs (Table 3). After adjusting for other baseline variables, CLI patients incurred additional costs €20,042 (*p* < 0.001) when compared with ALI, whereas AVI patients incurred significantly lower costs (-€14,152, *p* < 0.001). Age and previous history of heart failure were also found to be significant predictors of reduced overall costs over 5 years, which could be explained, as well as the lower costs incurred by AVI patients, by high case fatality rates (Fig. 2). Patients with diabetes mellitus incurred significantly higher overall costs (€11,804, *p* = 0.014) than those without the condition, partly reflecting the association with CLI.

**Table 3:** Baseline predictors of 5 year hospital care costs after an incident ACLVI event.

	Coefficient	Marginal effect	*P* > |z|
Age, years	−0.014	-€432	0.020
Gender (male)	−0.099	-€3062	0.450
Previous symptomatic vascular disease			
Coronary artery disease	0.005	€154	0.972
Heart failure	−0.391	-€12,039	0.018
Stroke or TIA	0.149	€4589	0.343
Stable PAD	−0.128	-€3956	0.411
Smoking history			
Never smoked	Reference case		
Previous smoker	−0.011	-€387	0.942
Current smoker	−0.501	-€13,827	0.007
Other risk factors			
Diabetes mellitus	0.383	€11,804	0.014
Hypertension	0.170	€5240	0.245
Atrial fibrillation	−0.073	-€2259	0.635
Hyperlipidaemia	0.163	€5026	0.271
Index of Multiple Deprivation	0.011	€336	0.189
Severity of ischaemia on admission	−0.247	-€7619	0.101
Incident ACLVI event:			
Acute limb ischaemia	Reference case		
Acute visceral ischaemia	−1.065	-€14,152	<0.001
Critical limb ischaemia	0.656	€20,042	<0.001
Constant	11.017		<0.001

#### Subsequent predictors

Average 5 year hospital care costs for the 89 patients who experienced one or more subsequent ACLVI event types after the incident event were €52,649, compared with €20,655 for those who did not (*p* < 0.0001, Table S4). By event type, patients with subsequent CLI had the highest mean costs at €62,508, as opposed to patients with subsequent ALI (€37,688; *p* = 0.026) and AVI (€23,339; *p* = 0.015).

Of the 280 patients with limb ischaemia, 230 (82%) had at least one endovascular or surgical intervention. A considerable proportion of patients underwent angioplasty or stenting (*n* = 154, 55%). Patients undergoing a surgical or endovascular intervention incurred significantly higher costs at 5 years than those who did not (Table S4).

For patients with limb ischaemia, after adjusting for the type of limb ischaemia event (i.e. ALI or CLI) and subsequent surgical interventions, subsequent ACLVI events did not significantly increase 5 year hospital care costs (Table 4). Endovascular and all surgical procedures (except for bypass surgery, endarterectomy, and laparotomy) following acute ALI or CLI significantly increased costs. For example, each additional below and above knee amputation increased costs by €17,406 (*p* < 0.001) and €25,692 (*p* < 0.001), respectively, over 5 years.

**Table 4:** Impact of subsequent interventions and ACLVI events on 5 year hospital care costs in patients with limb ischaemia.

	Coefficient	Marginal effect	*P* > |z|
Type of critical limb ischaemia			
Acute limb ischaemia	Reference case		
Critical limb ischaemia	0.706	€21,331	<0.001
Subsequent interventions			
Angioplasty/stenting	0.183	€6708	0.014
Bypass surgery	0.177	€6473	0.062
Embolectomy	0.636	€23,285	<0.001
Laparotomy^a^	0.620	€22,716	0.239
Endarterectomy	−0.078	-€2855	0.708
Below knee amputation	0.475	€17,406	<0.001
Above knee amputation	0.701	€25,692	<0.001
Subsequent ACLVI events			
Acute limb ischaemia	−0.254	-€9313	0.202
Acute visceral ischaemia	−0.366	-€13,410	0.172
Critical limb ischaemia	0.081	€2964	0.421
Constant	9.145		<0.001

a Performed in a patient with limb ischaemia who had a subsequent AVI.

### Benchmarking costs after an ACLVI event with those after stroke

Between April 2002 and March 2007, there was complete 5 year follow up for 793 patients with a first-in-study-period stroke. The mean 5 year total hospital care costs after stroke were €22,623 (SD 28,348), €6145 lower than those after an ACLVI event (95% CI difference: 1999–10,291; *p* = 0.004, Table 5). Patients with an ACLVI event had significantly higher hospital inpatient stay and outpatient costs than did stroke patients (*p* = 0.010 and *p* = 0.0004, respectively).

**Table 5:** Comparison of 5 year mean care costs (€, SD) after an incident ACLVI event and stroke.

	Emergency transport	Accident & Emergency	Day cases	Inpatient stays	Outpatient visits	Total hospital costs	Long-term institutionalisation	Total care costs
5 year mean care costs (€, SD)								
ACLVI events	252 (372)	174 (253)	1091 (3037)	25,068 (32,944)	2183 (4016)	28,768 (34,859)	6443 (30,468)	35,211 (50,500)
Stroke	295 (411)	193 (255)	851 (1505)	19,893 (27,594)	1390 (1819)	22,623 (28,348)	12,769 (41,401)	35,391 (55,755)
5 year mean care cost differences between ACLVI and stroke (€)								
Mean (95% CI)	−43 (−91–5)	−19 (−51–13)	240 (−95–575)	5175 (1230–9120)	793 (354–1232)	6145 (1999–10,291)	−6326 (−10,623–2023)	−180 (−6735–6375)
*p*>|z|	0.081	0.243	0.160	0.010	0.0004	0.004	0.004	0.957

Five year costs of long-term institutionalisation after stroke were €12,769 (SD 41,401), which was significantly higher than the 5 year institutionalisation costs after an ACLVI event (€6443; *p* = 0.004). Overall, when including hospital and long-term institutionalisation care costs, 5 year total care costs after an ACLVI event were similar to those after stroke (€35,211 vs. €35,391; *p* = 0.957).

## Discussion

Most previous studies assessing the healthcare costs of PAD have been based on hospital coding data of diagnosis or interventions.^5^, ^6^, ^7^ Although these types of study aim to estimate the costs of PAD in a large population, allowing them to individually link patients' medical records over time, they rely heavily on accurate coding to identify the index event. However, evidence from OXVASC has previously shown that, at least for the UK, the accuracy of routine medical coding is poor, with only 49% of ACLVI events correctly identified, and many events incorrectly classified as peripheral vascular disease.^3^ By contrast, OXVASC is the first ever large scale prospective study of all acute/critical limb or visceral ischaemia nested within a comprehensive, population based study of all acute vascular events without exclusion of patients by age or sex, including both hospitalisation and long-term institutionalisation costs.

The present study showed that the hospital care costs following an ACLVI event are considerable, with patients incurring 5 year total care costs of €35,211. It was found that patients with the more severe ACLVI events incurred the lowest costs, with patients with AVI incurring costs of €16,476, followed by ALI (€24,437), and highest costs after CLI (€50,814). With very high mortality rates shortly after the event, patients with AVI, and to a lesser degree ALI, were less likely to incur costs over the long-term. As the present study was undertaken using a long-term time perspective, it was possible to compare the costs incurred at each follow up year. This showed that over half of total costs (54%) were incurred over the first year following the incident ACLVI event.

Given that 5 year hospital care and long-term institutionalisation costs after stroke have previously been evaluated in the same patient population using the same methodology and unit costs to value resource use,^12^, ^13^ there was a unique opportunity to benchmark and compare the costs of ACLVI events with those of stroke in a meaningful way. It was found that 5 year hospital care costs after an ACLVI event were considerably higher than for stroke (€28,768 vs. €22,623, *p* = 0.004). Despite higher mortality rates than for stroke, the increased costs after ACLVI events are, in part, explained by the high rates of procedures in these patients. In OXVASC, 82% of patients with limb ischaemia underwent either endovascular or surgical procedures. As shown in the multivariate analyses, these interventions significantly increased costs over 5 years, with every above knee amputation increasing care costs by €25,692 in patients with limb ischaemia. By contrast, 5 year long-term institutionalisation costs were significantly lower after an ACLVI event than stroke (€6443 vs. €12,769; *p* = 0.004). Therefore, it was found that, overall, 5 year total care costs after an ACLVI event were comparable with those after stroke (€35,211 vs. €35,391; *p* = 0.957). Given that the costs of stroke have been widely assessed in a number of countries and settings,^20^ it is believed that these comparisons will be useful to other researchers and clinicians to help them better understand the likely economic consequences after an ACLVI event in their own setting.

Although the present study did not compare the care costs following stroke and an ACLVI event with those after myocardial infarction (MI) in OXVASC, previous studies have shown that the care costs of stroke are higher in the long term than those of MI.^21^, ^22^, ^23^, ^24^, ^25^ Given that costs of CLI are substantially higher than those for overall ACLVI events, this would therefore suggest that CLI is one of the costliest cardiovascular events.

In addition to previously noted limitations of the study,^14^ some further points are noteworthy. First, the study omitted other relevant healthcare costs such as those relating to primary care visits or community care. However, it is likely, as with stroke or other vascular disease, that these costs will represent only a small proportion of total healthcare costs.^20^ Second, costs of all ACLVI events were benchmarked against those of all stroke events, which is a relatively crude comparison, but overall costs are probably of most use given the very heterogeneous nature of both conditions. Third, by not recording the prevalence of patients with stable intermittent claudication, it was not possible to estimate the total clinical and economic burden of PAD, although all acute events in such patients were studied. Finally, the multivariate analyses performed to determine the predictors of 5 year hospital care costs included a large number of covariates, which were at the limits of the statistical power.

Current European guidelines on peripheral arterial disease summarise the evidence base for diagnosis and management of acute and critical limb ischaemia and acute visceral ischaemia, and identify gaps in evidence, pointing out that contemporary data on the epidemiology and “real world” outcomes of peripheral arterial disease are lacking.^26^ There is also a lack of data on the healthcare cost of peripheral arterial disease. The Oxford Vascular Study is the first prospective, population based study of all acute manifestations of vascular disease, including peripheral arterial diseases. It provides contemporary epidemiological data and highlights the poor outcome and considerable hospital care costs of peripheral arterial disease, which should inform health service provision, clinical decision making, and future research priorities.

The present results also highlight the considerable cost differences observed across ACLVI events, but also show that when taken together the hospital care costs after these events are greater than those after stroke and remained comparable even after long-term institutionalisation costs were taken into account.

## Conflict of interest

None.

## Funding

The Oxford Vascular Study has been funded by the Wellcome Trust (WT095626), Wolfson Foundation, Dunhill Medical Trust (OSRP2/1006), and the National Institute for Health Research (NIHR) Biomedical Research Centre, Oxford. PMR has a NIHR and Wellcome Trust Senior Investigator Award. The funding sources had no involvement with the study

## Appendix A Supplementary data

mmc1

## References

[1] Mozaffarian D., Benjamin E.J., Go A.S., Arnett D.K., Blaha M.J., Cushman M. (2016). Heart disease and stroke statistics – 2016: a report from the American Heart Association. Circulation.

[2] Leal J., Luengo-Fernandez R., Burns R., Wilkins E., Wilson L., Wickramasinghe K., Bhatnagar P., Leal J., Luengo-Fernandez R. (2017). Economic costs. European cardiovascular disease Statistics.

[3] Howard D.P.J., Banerjee A., Fairhead J.F., Hands L., Silver L.E., Rothwell P.M. (2015). Population-based study of incidence, risk factors, outcome, and prognosis of ischemic peripheral arterial events: implications for prevention. Circulation.

[4] Jaff M.R. (2014). Why patients know more about cars than peripheral artery disease. Circulation.

[5] Hirsch A.T., Hartman L., Town R.J. (2008). Virnig. National health care costs of peripheral arterial disease in the Medicare population. Vasc Med.

[6] Peacock J.M., Keo H.H., Duval S., Bamgartner I., Oldenburg N.C., Jaff M.R. (2011). The incidence and health economic burden of ischemic amputation in Minnesota, 2005-2008. Prev Chronic Dis.

[7] Currie C.J., Morgan C.L.L., Peters J.R. (1998). The epidemiology and cost of inpatient care for peripheral vascular disease, infection, neuropathy, and ulceration in diabetes. Diabetes Care.

[8] Alva M.L., Gray A., Mihaylova B., Leal J., Holman R.R. (2015). The impact of diabetes-related complications on healthcare costs: new results from the UKPDS (UKPDS 84). Diabet Med.

[9] Malone M., Lau N.S., White J., Novak A., Xuan W., Iliopoulos J. (2014). The effect of diabetes mellitus on costs and length of stay in patients with peripheral arterial disease undergoing vascular surgery. Eur J Vasc Endovasc Surg.

[10] Mahoney E.M., Wang K., Keo H.H., Duval S., Smolderen K.G., Cohen D.J. (2010). Vascular hospitalisation rates and costs in patients with peripheral artery disease in the United States. Circ Cardiovasc Qual Outcomes.

[11] Van Asselt A.D.I., Nicolai S.P.A., Joore M.A., Prins M.H., Teijink J.A.W. (2011). Cost-effectiveness of exercise therapy in patients with intermittent claudication: supervised exercise therapy versus a ‘Go Home and Walk’ advice. Eur J Vasc Endovasc Surg.

[12] Luengo-Fernandez R., Gray A.M., Rothwell P.M. (2012). A population-based study of hospital care costs during 5 years after transient ischemic attack and stroke. Stroke.

[13] Luengo-Fernandez R., Paul N.L., Gray A.M., Pendlebury S.T., Bull L.M., Welch S.J. (2013). Population-based study of disability and institutionalization after transient ischemic attack and stroke:10-year results of the Oxford Vascular Study. Stroke.

[14] Rothwell P.M., Coull A.J., Silver L.E., Fairhead J.F., Giles M.F., Lovelock C.E. (2005). Population-based study of event-rate, incidence, case fatality, and mortality for all acute vascular events in all arterial territories (Oxford Vascular Study). Lancet.

[15] Insall R.L., Davies R.J., Prout W.G. (1989). Significance of Buerger's test in the assessment of lower limb ischaemia. J R Soc Med.

[16] Department of Health. NHS reference costs 2013 to 2014. https://www.gov.uk/government/publications/nhs-reference-costs-2013-to-2014 (accessed 10 February 2017).

[17] Personal Social Services Research Unit. Unit Costs of Health & Social Care 2016. http://www.pssru.ac.uk/project-pages/unit-costs/unit-costs-2016/(accessed 10 February 2017).

[18] EUROSTAT. Database: Your key to European Statistics. http://ec.europa.eu/eurostat/data/database (accessed 10 February 2017).

[19] van Haelst S.T.W., Koopman C., den Ruijter H.M., Moll F.L., Visseren F.L., Vaartjes I. (2018). Cardiovascular and all-cause mortality in patients with intermittent claudication and critical limb ischaemia. Br J Surg.

[20] Luengo-Fernandez R., Gray A.M., Rothwell P.M. (2009). Costs of stroke using patient-level data: a critical review of the literature. Stroke.

[21] Nicholson G., Gandra S.R., Halbert R.J., Richhariya A., Nordyke R.J. (2016). Patient-level costs of major cardiovascular conditions: a review of the international literature. Clinicoecon Outcomes Res.

[22] Hallberg S., Gandra S.R., Fox K.M., Mesterton J., Banefelt J., Johansson G. (2016). Healthcare costs associated with cardiovascular events in patients with hyperlipidemia or prior cardiovascular events: estimates from Swedish population-based register data. Eur J Health Econ.

[23] Banefelt J., Hallberg S., Fox K.M., Mesterton J., Paoli C.J., Johansson G. (2016). Work productivity loss and indirect costs associated with new cardiovascular events in high-risk patients with hyperlipidemia: estimates from population-based register data in Sweden. Eur J Health Econ.

[24] Schmid T. (2015). Costs of treating cardiovascular events in Germany: a systematic literature review. Health Econ Rev.

[25] Punekar R.S., Fox K.M., Richhariya A., Fisher M.D., Cziraky M., Gandra S.R. (2015). Burden of first and recurrent cardiovascular events among patients with hyperlipidemia. Clin Cardiol.

[26] Aboyans V., Ricco J.B., Bartelink M.E.L., Björck M., Brodmann M., Cohnert T. (2018). 2017 ESC guidelines on the diagnosis and treatment of peripheral arterial diseases, in collaboration with the European society for vascular surgery (ESVS). Eur J Vasc Endovasc Surg.

