# Supplementary material for: Hospital and Institutionalisation Care Costs after Limb and Visceral Ischaemia Benchmarked Against Stroke: Long-Term Results of a Population Based Cohort Study
Source: Eur J Vasc Endovasc Surg. 2018 Aug;56(2):271–81. doi: 10.1016/j.ejvs.2018.03.007 (PMC6105571; doi:10.1016/j.ejvs.2018.03.007)
Supplement: mmc1 [file mmc1.docx]

**Online supplementary appendix**

**Appendix 1. Study population and case ascertainment**

The OXVASC study population comprises all individuals (10-year average, 92,728), regardless of age, registered with ≈100 family physicians in 9 primary care practices in Oxfordshire, UK.^1,2^ In the United Kingdom, the vast majority of individuals register for primary health care, which provides a lifelong record of all medical consultations and details of medications, BP measurements, and investigations. All participating practices held accurate age/sex patient registers and allowed regular searches of their computerized diagnostic coding systems. All practices refer patients to only 1 major secondary care center.

The OXVASC study population is 94% white, 3.1% Asian, 1.5% Chinese, and 1.4% Afro-Caribbean.^2^ On the basis of the Index of Multiple Deprivation,^3^ the electoral wards covering our population are less deprived than the rest of England (mean Index of Multiple Deprivation score, 8.69 versus 16.98; t test, p<0.001) but have a broad range of deprivation, with 22% of wards ranking in the lower third nationally. OXVASC was approved by our local research ethics committee.

Case ascertainment was by prospective daily searches for acute events in hospital (hot pursuit), supplemented by searches of discharge and primary care diagnostic coding data (cold pursuit). Hot pursuit was based on the daily assessment of all patients with a possible vascular event identified by: 1) Daily searches of Emergency Department admission and symptom/diagnosis registers; 2) Daily listing from the central admissions department of all patients from our general practices admitted to hospital, and assessment of these patients in hospital; 3) Daily visits to the cardiac surgery and vascular surgery wards and review of daily lists of all patients referred to vascular surgery; 4) Daily identification via Bereavement Officers of patients dead on arrival at hospital or who died soon after; 5) Daily assessment of all patients undergoing diagnostic angiographic, angioplasty/stenting or arterial surgical procedures in any territory.

The methods of cold pursuit were: 1) Weekly review of all listed surgical procedures undertaken by vascular and cardiovascular surgery; 2) Frequent contact with general practices and monthly searches of computerised practice diagnostic codes; 3) Monthly practice-specific list of all patients with relevant diagnostic codes from the coding departments covering all acute and community hospitals (Hospital Episode Statistics – HES data); 4) Monthly visits to the Coroner’s Office to review out-of-hospital deaths; 5) Review of all death certificates and relevant clinical details in the study practices; 6) Practice-specific listings of all ICD-10 death codes from the local Department of Public Health; 7) Review of vascular surgery outpatient clinic letters to identify patients who were not admitted to hospital. For all cases not initially identified by HES data and death certification, these data sources were re-searched using NHS number and other identifiers, where possible.

A study clinician assessed patients acutely in hospital (or at home if ascertained late). Informed consent was sought when possible, or assent was obtained from a relative. Standardized clinical history and examination were recorded, with details of medication, history, and all investigations and interventions occurring subsequent to the event. All diagnoses were reviewed by a vascular surgeon. If a patient died before assessment or was identified only by cold pursuit, eyewitness accounts were obtained and relevant records reviewed. If death occurred outside the hospital or before investigation, autopsy results were reviewed. Clinical details were sought from primary care physicians or other clinicians on all deaths resulting from a possible vascular cause.

Cardiovascular examination included assessment of the peripheral pulses, the Buerger test, and absolute ankle pressure and anklebrachial pressure index recordings. The Buerger's test is an assessment of arterial sufficiency and is of diagnostic value in CLI. The vascular angle, which is also called Buerger's angle, is the angle to which the leg has to be raised before it becomes pale, whilst in supine decubitus. In a limb with a normal circulation the toes and sole of the foot, stay pink, even when the limb is raised by 90 degrees. In an ischaemic leg, elevation to 15 degrees or 30 degrees for 30 to 60 seconds may cause pallor. A vascular angle of less than 20 degrees indicates severe ischaemia. From a sitting position, in normal circulation, the foot will quickly return to a pink colour. Where there is peripheral artery disease the leg will revert to the pink colour more slowly than normal and also pass through the normal pinkness to a red-range colouring (rubor - redness). This is due to the dilatation of the arterioles to rid the metabolic waste that has built up in a reactive hyperaemia. Finally, the foot will return to its normal colour.

For patients with incompressible ankle signals, pressures were estimated by pole test. For patients in whom clinical vascular assessment was not possible by the study clinician before urgent revascularization or death, the assessments made by the admitting clinician were used.

All survivors were followed up by a research nurse at 6 months and subsequently by their family doctor, with recurrent events also identified by the ongoing study ascertainment. If a vascular event was suspected, the patient was reassessed by a study physician. Premorbid disability and disability on follow-up were assessed with the modified Rankin Scale.^4^

To assess key vascular risk factors and levels of premorbid control, we extracted individual data from primary healthcare records for all cases and compared these data with group average data for the underlying unaffected population. The accuracy of these data was tested in cases through direct questioning of patients and relatives and review of records. All premorbid BP measurements were extracted from primary care records of incident cases. BP was generally taken with automated sphygmomanometers by a primary care physician or practice nurse. A cutoff value of 140/90 mmHg was used to define hypertension.

All patients with an acute/critical limb or visceral ischaemia (ACLVI) event from April 1, 2002, to March 31, 2012, from the study registered practices were included. Events were defined as any acute arterial event that affected a limb or an organ other than the heart or the brain/eye and led to hospital assessment/admission or caused death in the community. The likely cause (atherosclerotic/in situ thrombosis, embolic, diabetic microvascular disease, iatrogenic, graft/stent thrombosis, or multifactorial) was determined by a vascular surgeon taking into account clinical findings and subsequent investigations.

ALI was defined as an arterial event of sudden onset and <2 weeks in duration resulting in symptomatic limb ischemia.^5^ Degree of limb ischemia was graded by the Rutherford classification of severity as viable, threatened-marginal, threatened-immediate, or irreversible.^6^ AVI was defined as acute arterial events of sudden onset and <2 weeks in duration resulting in symptomatic visceral ischemia (including bowel, liver, spleen, and renal end-organ compromise). Severity of ischemia was graded by the presence or absence of lactic acidosis on admission. CLI has several overlapping diagnostic classification systems.^5-8^ The original Fontaine and Rutherford classification systems describe symptoms persisting for >2 weeks and delineate intermittent claudication from ischemic rest pain and ulceration. More recent defining criteria from the Trans-Atlantic Inter-Society Consensus (TASC) steering committee^5^, Society of Vascular Surgery (United States),^6^ and European Union,^8^ require both clinical and objective assessment of absolute ankle pressure, ankle-brachial pressure index, or toe pressure (eg, plethysmographic or laser Doppler techniques). As pointed out in the latest TASC consensus statement,^5^ strict objective criteria exclude some patients with imminently threatened ischemic limbs in need of urgent revascularization. We therefore included all patients whose symptoms had been present for >2 weeks with ischemic rest pain or tissue loss of sufficient severity to warrant urgent hospital admission and thought to be secondary to large- or small-vessel arterial disease. Objective measurements, although performed, were not required for inclusion. Incidence was compared on the basis of all current diagnostic criteria.

**References**

1. Rothwell PM, Coull AJ, Silver LE, Fairhead JF, Giles MF, Lovelock CE, et al. Population-based study of event-rate, incidence, case fatality, and mortality for all acute vascular events in all arterial territories (Oxford Vascular Study). Lancet. 2005;366:1773–1783.
2. Office for National Statistics. 2001 and 2011 Census area statistics. <http://www.ons.gov.uk/ons/guide-method/census/2011/index.html>. (Accessed 10 February 2017)
3. UK Department for Communities and Local Government. English indices of deprivation 2000 and 2010. <https://www.gov.uk/government/publications/english-indices-of-deprivation-2010>. (Accessed 10 February 2017)
4. Farrell B, Godwin J, Richards S, Warlow C. The United Kingdom Transient Ischaemic Attack (UK-TIA) aspirin trial: final results. J Neurol Neurosurg Psychiatry. 1991;54:1044–1054.
5. Norgren L, Hiatt WR, Dormandy JA, Nehler MR, Harris KA, Fowkes FG; TASC II Working Group. Inter-society consensus for the management of peripheral arterial disease (TASC II). J Vasc Surg. 2007;45(suppl S):S5– S67.
6. Rutherford RB, Baker JD, Ernst C, Johnston KW, Porter JM, Ahn S, et al. Recommended standards for reports dealing with lower extremity ischemia: revised version. J Vasc Surg. 1997;26:517–538.
7. Anderson JL, Halperin JL, Albert NM, Bozkurt B, Brindis RG, Curtis LH, et al. Management of patients with peripheral artery disease (compilation of 2005 and 2011 ACCF/AHA guideline recommendations): a report of the American College of Cardiology Foundation/ American Heart Association Task Force on Practice Guidelines. Circulation. 2013;127:1425–1443.
8. Second European consensus document on chronic critical leg ischaemia. Eur J Vasc Surg. 1992; 6:1–28.

**Supplementary Table I** 5-year mean discounted care costs (€, S.D.) after incident ACLVI event

|  | Emergency transport | Accident & Emergency | Day cases | Inpatient stays | Outpatient visits | Total hospital costs | Long-term institutionalisation | Total care costs |
| --- | --- | --- | --- | --- | --- | --- | --- | --- |
| All events | 240 (352) | 166 (239) | 1,038 (2,839) | 24,370 (31,840) | 2,103 (3,776) | 27,917 (33,583) | 5,899 (27,857) | 33,815 (47,570) |
|  | | | | | | | | |
| ALI | 201 (307) | 139 (210) | 775 (1,258) | 16,171 (28,113) | 1,529 (2,150) | 18,815 (29,117) | 4,607 (26,717) | 23,422 (41,967) |
| AVI | 208 (313) | 137 (196) | 542 (1,272) | 6,706 (12,543) | 514 (1,122) | 8,107 (13,900) | 7,382 (35,923) | 15,489 (43,677) |
| CLI | 268 (380) | 187 (262) | 1,322 (3,583) | 34,009 (34,414) | 2,903 (4,605) | 38,689 (35,939) | 5,895 (25,013) | 44,584 (48,300) |

ANOVA test for differences in total costs in acute peripheral event groups at 5 years <0.0001

**Supplementary Table II.** Baseline characteristics

|  | **All**  **n (%)** | **ALI n (%)** | **AVI n (%)** | **CLI n (%)** |
| --- | --- | --- | --- | --- |
| Total | 351 (100) | 81 (100) | 71 (100) | 199 (100) |
| Mean (SD) age, years | 76 (12) | 76 (12) | 79 (15) | 75 (11) |
| Gender (males) | 174 (50) | 41 (51) | 26 (37) | 107 (54) |
| Previous symptomatic vascular disease |  |  |  |  |
| Coronary artery disease | 121 (35) | 22 (28) | 24 (34) | 75 (38) |
| Heart failure | 76 (22) | 15 (19) | 21 (30) | 40 (20) |
| Stroke or TIA | 77 (22) | 21 (26) | 15 (21) | 41 (21) |
| Stable peripheral arterial disease | 177 (50) | 27 (33) | 10 (14) | 140 (70) |
| Risk factors |  |  |  |  |
| Ever smoked | 241 (70) | 55 (71) | 46 (67) | 140 (71) |
| Diabetes mellitus | 102 (29) | 7 (9) | 7 (10) | 88 (44) |
| Hypertension | 236 (67) | 46 (57) | 49 (69) | 141 (71) |
| Atrial fibrillation | 99 (28) | 32 (40) | 26 (37) | 41 (21) |
| Hyperlipidaemia | 164 (47) | 23 (28) | 31 (44) | 110 (56) |
| Mean (SD) Index of Multiple Deprivation | 11 (8) |  |  |  |
| Most deprived – 1^st^ quintile | 13 (4) | 3 (4) | 2 (3) | 8 (4) |
| 2^nd^ quintile | 15 (4) | 1 (1) | 2 (3) | 12 (6) |
| 3^rd^ quintile | 66 (19) | 15 (19) | 13 (18) | 38 (19) |
| 4^th^ quintile | 115 (33) | 32 (40) | 22 (31) | 61 (31) |
| Least deprived - 5^th^ quintile | 142 (40) | 30 (37) | 32 (45) | 80 (40) |

Supplementary Table III. Univariate baseline predictors of 5-year total hospital costs (€)

|  | **Mean 5-year costs (S.D.)** | **p>\|z\|** |
| --- | --- | --- |
| Age <65 years | 31,031 (37,412) | 0.027 |
| Age 65 to 74 years | 29,891 (33,813) |  |
| Age 75 to 84 years | 33,939 (38,815) |  |
| Age >85 years | 19,696 (26,330) |  |
| Male | 31,089 (37,890) | 0.217 |
| Female | 26,486 (31,540) |  |
| No history of coronary artery disease | 27,648 (34,996) | 0.392 |
| History of coronary artery disease | 31,008 (34,756) |  |
| No history of heart failure | 30,130 (33,807) | 0.164 |
| History of heart failure | 23,838 (38,261) |  |
| No history of stroke or TIA | 27,444 (32,689) | 0.180 |
| History of stroke or TIA | 33,479 (41,580) |  |
| No history of stable PAD | 22,176 (31,161) | 0.0004 |
| History of stable PAD | 35,248 (37,106) |  |
| Never smoked | 30,880 (32,778) | 0.231 |
| Previous smoker | 31,400 (40,474) |  |
| Current smoker | 23,736 (26,198) |  |
| No diabetes mellitus | 21,013 (24,402) | <0.0001 |
| Diabetes mellitus | 47,799 (47,308) |  |
| No hypertension | 25,574 (34,798) | 0.231 |
| Hypertension | 30,324 (34,857) |  |
| No atrial fibrillation | 30,575 (36,033) | 0.121 |
| Atrial fibrillation | 24,169 (31,379) |  |
| No hyperlipidaemia | 22,496 (24,675) | 0.0003 |
| Hyperlipidaemia | 35,939 (42,674) |  |
| Deprivation by postcode of residence |  | 0.042 |
| Most deprived – 1^st^ quintile | 26,767 (22,558) |  |
| 2^nd^ quintile | 50,475 (60,018) |  |
| 3^rd^ quintile | 35,057 (37,013) |  |
| 4^th^ quintile | 27,050 (36,715) |  |
| Least deprived - 5^th^ quintile | 25,126 (28,447) |  |
| Severity of ischaemia on admission |  | 0.096 |
| Non-severe | 24,304 (33,574) |  |
| Severe | 30,915 (35,330) |  |

**Supplementary Table IV**. Univariate subsequent predictors of 5-year total hospital costs (€)

|  | **n** | **Mean 5-year costs (S.D.)** | **p>\|z\|** |
| --- | --- | --- | --- |
| **All patients** | | | |
| No subsequent ACLVI events | 262 | 20,655 (26,183) |  |
| ≥ 1 subsequent ACLVI events | 89 | 52,649 (45,023) | <0.0001* |
|  |  |  |  |
| ≥ 1 subsequent ALI | 18 | 37,668 (30,915) | 0.009* |
| ≥ 1 subsequent AVI | 11 | 23,339 (24,519) | 0.727* |
| ≥ 1 subsequent CLI | 66 | 62,508 (47,295) | <0.0001* |
| **Patients with limb ischaemia** | | | |
| No subsequent intervention related to limb ischaemia | 50 | 9,169 (13,646) |  |
| ≥ 1 subsequent intervention related to limb ischaemia | 230 | 39,319 (37,775) | <0.0001† |
|  |  |  |  |
| ≥ 1 angioplasty/stent | 154 | 41,987 (40,993) | <0.0001† |
| ≥ 1 bypass surgery | 63 | 46,410 (33,863) | <0.0001† |
| ≥ 1 embolectomy | 41 | 32,933 (41,049) | <0.0001† |
| ≥ 1 endarterectomy | 14 | 45,889 (45,777) | <0.0001† |
| ≥ 1 below knee amputation | 66 | 62,443 (48,911) | <0.0001† |
| ≥ 1 above knee amputation | 35 | 66,538 (41,716) | <0.0001† |

*When compared to patients with no subsequent ACLVI events

†When compared to patients with no subsequent interventions
